# Feasibility of Using Wearables for Home Monitoring during Radiotherapy for Head and Neck Cancer—Results from the OncoWatch 1.0 Study

**DOI:** 10.3390/cancers15020422

**Published:** 2023-01-09

**Authors:** Cecilie Holländer-Mieritz, Emma Balch Steen-Olsen, Claus Andrup Kristensen, Christoffer Johansen, Ivan Richter Vogelius, Helle Pappot

**Affiliations:** 1 Department of Oncology, Centre for Cancer and Organ Diseases, Copenhagen University Hospital-Rigshospitalet, 2100 Copenhagen, Denmark; 2 Department of Clinical Medicine, Faculty of Health and Medical Sciences, University of Copenhagen, 2200 Copenhagen, Denmark

**Keywords:** wearables, smartwatch, home monitoring, patient-generated health data, radiotherapy, head and neck cancer

## Abstract

**Simple Summary:**

Wearables such as consumer smartwatches allow the home monitoring of, e.g., heart rate and physical activity, which may play a role in the clinical care of patients with cancer. Patients with head and neck cancer often experience side effects when they receive curatively intended radiotherapy and optimization of symptom monitoring is wanted. In general, the research on wearables for clinical care and symptom monitoring is limited but evolving. Prior studies have primarily used wrist-worn activity trackers and not the more advanced smartwatches. In the OncoWatch 1.0 study, we investigated the feasibility of using a smartwatch for monitoring heart rate and physical activity. Although adherence to using the smartwatch was lower than expected, we successfully established and tested a secure data setup and identified key elements to be considered in future studies.

**Abstract:**

Background: Consumer wearables allow objective health data monitoring, e.g., of physical activity and heart rate, which might change over a cancer treatment course. Patients with head and neck cancer (HNC) receiving radiotherapy (RT) with curative intent typically experience side effects such as pain, decreased appetite, and dehydration, which may lead to hospitalizations. Therefore, health data monitoring could be important to understand a patient’s condition outside the hospital. The OncoWatch 1.0 study investigated the feasibility of using smartwatches for patients with HNC receiving RT. Methods: This study was a prospective, single-cohort feasibility study. The inclusion criteria were patients ≥ 18 years of age who planned to receive curatively intended radiotherapy for HNC. Consenting patients were asked to wear a smartwatch during RT and until two weeks after the end of RT. The primary endpoint was adherence. The secondary endpoints were data acquisition and variations in heart rate and physical activity. Results: Ten patients were included, with a median age of 62 years and eight males. The adherence rate for wearing the watch >12 h/d over the study period was 31%. The data acquisition rate was 61%. Conclusions: Although the primary endpoint was not reached, new knowledge has been established, including the secure data setup and key points that need to be addressed in future studies.

## 1. Introduction

Radiotherapy (RT) or chemoradiotherapy (CRT) for head and neck cancer (HNC) is known to cause acute side effects such as pain, dysphagia, decreased appetite, and fatigue [1,2]. These symptoms often lead to a deterioration in the patient’s health condition, affecting the patient’s quality of life (QoL) during the six–seven weeks RT period [3]. The degree of acute side effects is also associated with unplanned hospital encounters and admissions [4]. Increased symptom monitoring, enabling symptom handling and leading to better QoL, is needed in the HNC population [5,6]. There is an increased focus on home monitoring to help patients manage their symptoms and take some pressure off hospitals. Patient-Generated Health Data (PGHD) can provide valuable information about a patient to healthcare professionals [5,7]. One type is Patient Reported Outcomes (PRO), which provide information about a patient’s subjective symptoms and can be used for symptom monitoring or quality-of-life assessments [5]. Another type of PGHD is biometric sensor data, which can be collected with noninvasive wearables such as a smartwatch or an activity tracker [8,9]. Although consumer wearables are becoming more common in the general population, their use in a healthcare setting is still at an early stage [8,10]. Most studies using wearable technology in oncology have used wrist-worn wearables mainly to monitor physical activity [10]. Outcomes from the wearables also allow for the monitoring of physiological parameters, such as heart rate, respiration, and skin temperature [11,12]. Home monitoring with a wearable might provide hospitals with objective parameters that previously have only been available if a patient was admitted [13]. Vulnerable patients whose performance status allows them to receive antineoplastic treatment but who need extra support might benefit from closer home monitoring with minimal effort for the patient. Previously, Peterson et al. showed that HNC patients could be home-monitored with a stationary device monitoring weight, blood pressure, and heart rate. Patients who sent their daily monitored weight and blood pressure to their clinician had less severe symptoms [14,15]. In that study, the patients actively had to record the parameters. New technology, such as wearables, allows for passive monitoring. Recently, Sher et al. showed that head and neck cancer patients with a decrease in step count the previous week had a significant decrease in QoL [16]. However, the primary endpoint, which was 90% compliance with using the device, was not met. Thus, acquiring knowledge on adherence and practical feasibility in the healthcare setting is important when introducing new technologies [17]. Peterson et al.’s and Sher et al.’s were feasibility/pilot studies, while Ohri et al.’s was a pooled data analysis from three prospective clinical trials.

This feasibility study aimed to determine the adherence to using a smartwatch during curatively intended RT for HNC. Secondly, we report on data acquisition and the variations in heart rate and physical activity throughout the treatment course.

## 2. Materials and Methods

### 2.1. Design and Patients

The OncoWatch 1.0 study was a prospective, single-cohort feasibility study investigating the adherence to wearing a smartwatch and exploring the changes in heart rate and physical activity over the treatment course (ClinicalTrials.gov, NCT04613232). The study period was from inclusionuntil two weeks after RT. Consenting patients were asked to wear a smartwatch daily, preferably over 12 h per day, during the study period. In addition, biometric sensor data and demographic information were collected.

The inclusion criteria were patients aged ≥18 years and who planned to receive primary or postoperative curatively intended radiotherapy for HNC (5–6 fractions/week/up to 34 fractions) at the Department of Oncology, Rigshospitalet (Copenhagen, Denmark). Other inclusion criteria were the ability to read and speak Danish and having no severe cognitive deficits.

The study followed the General Data Protection Regulation (GDPR) and was approved at the Capital Region of Denmark (ID. PP-2019-797). The study was approved by the local division of IT and Medico Technology in the Capital Region of Denmark. According to Danish legislation, this study’s approval from the National Committee on Health Research Ethics was not required.

The patients received verbal and written information. Written informed consent was required, which informed patients that it was possible to withdraw from the study at any time during the study period. The hospital supplied the smartwatches and smartphones.

Further details of the protocol for the OncoWatch 1.0 study can be assessed in the published protocol article [18].

### 2.2. Device

The wearable used was a smartwatch type Apple Watch^®^ Series 5. The smartwatch was connected to an iPhone 8. The smartwatch and smartphone were supplied by the hospital and returned after the study termination. Based on IT security and safety discussions, the devices were limited to serve the functionality of the project and offered only rudimentary functionality apart from this.

### 2.3. Software

The OncoWatch app by ZiteLab ApS collected data from the Apple HealthKit and sent them to a secure, approved cloud server. The app was only available for study participants. A study assistant assigned each patient an account. Heart rate and step count data were extracted specifically for this study, although many other standard Apple HealthKit data were recorded and stored.

### 2.4. Variables

The primary endpoint was adherence to determine the feasibility of using a smartwatch. The research group defined adherence in this study as the number of patients who could wear the device at least 12 h per day during the study period. Wear time per day (hours) was defined as follows: Any clock hour in which there was at least one registration of heart rate or step count was recorded as “watch worn”. Hours without these recordings registered were recorded as “Watch not worn”. We then counted the number of hours of “watch worn” per day during the study period. The total study period was defined as 60 days. The adherence rate was the percentage of wear time >12 h per day during the study period. Secondary explorative endpoints were data acquisition events and variations in heart rate and physical activity (step counts). Data acquisition was defined as any data registration event on the smartwatch per day. The data acquisition rate was the percentage of data registrations during the study period. Since the watches were not worn 24 h/day (h/d), the heart rate measurements were averaged using only hours with “watch worn”, and the steps were normalized to the number of hours with “watch worn”, as per the previous definition. Clinical data from all patients, including age, stage, treatment regimen, admissions, and objective toxicity scores, were collected from the Danish head and neck cancer group (DAHANCA) database [19]. A screening log was completed to document recruitment status. Data were captured in REDCap. Data were analyzed using R-statistics.

## 3. Results

During the recruitment period from 22 January to 1 December 2021, 63 patients, 48 men and 15 women aged 30–82 years, were screened for enrollment in the OncoWatch 1.0 study. Twenty-seven of the sixty-three patients were never asked due to either the healthcare professionals’ assessment (*n* = 3), no reason for missing information (*n* = 11), or competing protocols (*n* = 13). A total of 23/63 patients declined participation, primarily with the reason that they could not oversee participation. Three of the sixty-three patients had disseminated disease that had not been identified initially.

Ten patients were included with a median age of sixty-two years and of which eight were males, as shown in Table 1. Nine patients finished curatively intended RT. One treatment plan was converted to palliative treatment, but the patient was retained in the analyses because it was still a long radiotherapy course. Only one patient was admitted to hospital during the study period. The patient needed a feeding tube upfront and was admitted for two days. Objective toxicity data were extracted from the DAHANCA database. However, the data were incomplete and not systematically obtained due to the COVID-19 pandemic.

### 3.1. Feasibility and Data Acquisition

Wear times of >12 h/d during the study period varied between 0–55 days, with a median of 5, as shown in Table 2. Only 2 patients had a wear time of >12 h/d for 90% of the study period. The total adherence rate for wearing the watches for >12 h/d over the study period was 31%. Days with any data registration varied between 4–59 (33, 5). Four patients had their last data registration before their treatment course ended. These patients had experienced technical difficulties or felt it was too demanding to charge and wear the smartwatch. The total data acquisition rate over the study period was 61%.

### 3.2. Biometric Data Outcome

**Figure 1:**
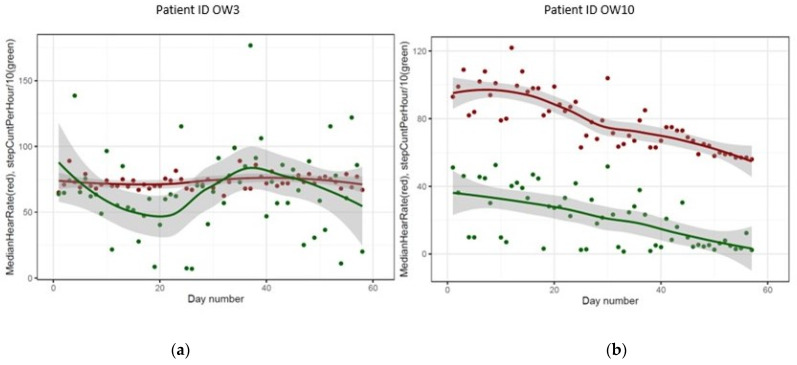
Examples of heart rate and step count variations during the study period: (**a**) patient ID OW3; (**b**) patient ID OW10.

The entire data file of the study provided 196,389 data points on 10 patients. Due to the low adherence, descriptive statistics for heart rate and step count were only analyzed for the four patients with the best compliance to explore the feasibility of defining and interpreting the outcome data. We observed mixed signals across the four patients. Two examples are shown in Figure 1. Plots for the remaining two patients are shown in Supplementary Figure S1. One patient, ID OW10, had a declining heart rate over time, which turned out to be correlated with declining activity over the course, as shown in Figure 1. While this could be interpreted as a sign of deterioration in the patient’s performance status, given the range of measurements and variability between patients, the data should be interpreted with caution and viewed as examples of the display. Another example (patient ID OW3) is illustrated in Figure 1. In this figure, heart rates and step counts remain in the same areas during the study period.

## 4. Discussion

In the OncoWatch 1.0 study, we established a framework for testing a smartwatch for home monitoring in a public healthcare hospital. In this study, we could not obtain the adherence rate to the smartwatch predefined in the study protocol [18]. Nevertheless, we gained novel knowledge on using smartwatches for collecting objective data in a patient group with HNC that is underrepresented in the studies of [10,20]. In addition, we established and successfully tested a secure setup for collecting objective data from a smartwatch that were transferred to a secure server. Studies using wrist-worn wearables in oncology during treatment have primarily used an activity tracker and not a smartwatch [10]. An activity tracker tracks only health and exercise, whereas a smartwatch allows for more functions, such as notifications, updates, and interactive use.

Before initiating the current study, a study protocol was published in a peer-reviewed journal [18]. The adherence rate (wear time >12/h/d during the study period) of 31% was lower than we had anticipated. Even though the primary endpoint was not met, this feasibility study has revealed several points. First, since this was a study in a public healthcare setting paid by taxes, all patients should be able to participate despite their socioeconomic situation. Secondly, since this was the first study using smartwatches for objective monitoring of patients in active cancer treatment outside the Hospital in Denmark, the authorities did not allow patients to use their own devices because of general data protection regulations. For that reason, the hospital supplied the smartwatches and connected phones. The phones had a secure setup and could only be used in this study. The initial choice of the smartwatch was precisely to test this device and not just a less advanced activity tracker, both of which are common consumer wearables. The smartwatch and its integrated functions can collect PROs directly on the watch or in a connected app. We hypothesized that the integrated functions for collecting health data could play a role in symptom management in the long term. However, we achieved a data acquisition rate of 61% over the study period. A systematic review of adherence and feasibility during cancer treatment across cancer types found that adherence varied from 60 to 100%. However, across all studies, there were different definitions of wear time per day and durations of the study period [10]. Furthermore, none of the studies reported in the review included patients with head and neck cancer as the primary diagnosis group. Curatively intended radiotherapy is a six–seven-week-long treatment course with planned visits five–six times per week, which is time-consuming and causes both acute and late side effects, which can also be a factor in the relatively low wear times and adherence rate.

Previous electronic PRO (ePRO) studies have shown that high compliance and adherence can be achieved [21]. Moreover, the advantage and prominent use of the smartwatches would be to have PRO questions sent to either the smartwatches or the connected app on the phones. Ensuring direct feedback from healthcare professionals to the patients would presumably enhance their incitement to use the devices. Similarly, the feedback to the patients and staff allocation has been shown to be important for the outcomes in ePRO studies [22]. We suspect that the task of charging the watch and the lack of personal use may have caused the patients to stop wearing the devices. For the specific Apple Watch model used, there was a need for almost daily charging. Newer models or other types of consumer wearables could decrease this issue. For a wearable to be practically feasible, it would probably have to be less demanding to use, have meaningful functions for the patient, or be integrated and used with the patients’ own devices. One would also suspect that non-adherence would be a particular problem in frail patients needing extra monitoring [23].

The current study had a low recruitment rate, which might, to some extent, have been affected by the COVID-19 pandemic [24,25], as well as a lack of resources to participate in a study using new technologies. One-third of patients declined inclusion primarily because they could not oversee participation because they would find it too demanding. Our experience from other studies in this patient group is that around half of the patients will decline participation primarily because they are exhausted, overwhelmed by the treatment regimen, or a lack of personal resources [2,26]. A healthcare professional’s incentive to ask a patient to participate can be affected by several causes, e.g., the individual healthcare professional’s opinion on whether they find the patient suited for the project, their knowledge of new technologies, a busy work program, or competing protocols [27].

Despite the low adherence, the entire data file of the study provided 196,389 data points on 10 patients. The predefined small study size only allowed for biometric data to be analyzed descriptively and only to describe an example of how data outcomes can be presented. A dataset of such complexity could yield highly significant false positive associations; therefore, this type of analysis was omitted. In future studies, it could be relevant to examine changes in daily step counts and heart rate over the course of a day [28]. Recently, Sher et al. showed that head and neck patients with a decrease in step count the previous week had a significant decrease in QoL [16]. However, the study found no association between changes in heart rate and hospital admissions or QoL. The patients in this study also experienced difficulties with compliance to the devices. Previously, Ohri et al. showed, in a study including patients with locally advanced NSCLC receiving definitive concurrent chemoradiotherapy, that inactive patients, based on step counts, were more likely to be hospitalized [29]. A recent scoping review by Huang et al. showed that out of 38 studies, both during and after cancer treatment and in mixed diagnosis groups, only 10 compared PROs with clinical outcomes. Eight indicated a positive correlation between the PROs and wearable outcomes [20]. As shown in our previous review, they also found that definitions of the outcome measures and adherence varied across the studies [10,20]. These and other studies [30,31] indicate that there might be a benefit from using wearables in oncology, but also that larger clinical studies confirming this are required.

Initiatives on selecting endpoints have been made, including the Clinical Transformation Initiative (CTTI) [32]. However, there is a need for guidelines on reporting endpoints, including adherence, to ensure standardized measures that can be compared across studies. It could be beneficial to draw on the experiences from the international guidelines for PRO reporting [22]. When designing, conducting, interpreting, and reporting the studies, one must remember that although consumer wearables are well known, they are not necessarily designed to be applied to the healthcare system and are still at an early testing stage in many areas. Therefore, based on our experiences from this study, we recommend that future studies include electronic PROs, qualitative interviews with patients and healthcare professionals, and the involvement of patients in the study designs.

## 5. Conclusions

The OncoWatch 1.0 study was a novel study investigating the use of smartwatches for the home monitoring of objective parameters in patients with head and neck cancer. Although the study did not reach its primary endpoint (adherence to using a smartwatch for over 12 h per day during the study period), new knowledge has been established and adds to the spare research in the field, including the testing of the secure data setup and challenges that need to be addressed in future studies. Patient-generated health data, including sensor data, are something the healthcare system cannot evade. Therefore, we recommend that knowledge from the feasibility studies be considered when designing longitudinal studies for home monitoring. PGHD can potentially provide relatively low-cost data of clinical relevance to improving treatment trajectories for patients undergoing RT.

## Figures and Tables

**Table 1:** Patient characteristics.

Characteristics	No.
Age, years, median	62 (50–75)
Gender	
Male	8
Female	2
Site	
Pharynx	2
Larynx	2
Oral cavity	5
Other	1
HPV/p16 status	
Positive	1
Negative	6
Unknown	3
Treatment modality	
CRT	1
RT	3
Postoperative CRT	1
Postoperative RT	5
Dose, Gy total	
66–68	8
56–60	2
Admissions	
Planned	1
Acute	0
Marital status	
Married/living with a partner	4
Single/divorced/living alone	6

CRT: chemoradiotherapy; RT: radiotherapy.

**Table 2:** Wear times and data acquisition.

Patient ID	Age	Gender	Civil Status	Tumor Site and Stage	Treatment Modality	Wear Time > 12 h/d (days)	Wear Time > 12 h/d (%)	Data Acquisition (days)	Data Acquisition (%)
OW10	75	F	Living alone, retired	Oral cavity, T2N0M0	Postoperative RT, 2 Gy × 33, 6 F/W	55	92	59	98
OW2	68	M	Divorced, living alone, retired	Oral cavity, T4aN0M0	Postoperative RT, 2 Gy × 30, 5 F/W	54	90	57	95
OW3	50	M	Married, working	Oral cavity, T4aN0M0	Postoperative RT, 2 Gy × 33, 6 F/W	42	70	58	97
OW9	50	M	Married, working	Gl. Parotitis, T2N0M0	Postoperative RT, 2 Gy × 33, 5 F/W	24	40	49	82
OW6	56	F	Married, working	Pharynx, T1N2bM0	CRT, 2 Gy × 33, 6 F/W	8	13	16	27
OW4	66	M	Married, working	Larynx, T2N0M0	RT, 2 Gy × 33, 6 F/W	1	2	18	30
OW7	56	M	Divorced, living alone, working	Larynx, T2N2cM0	RT, 4 Gy × 14, 2 F/W	0	0	4	7
OW8	74	M	Divorced, living alone, retired	Oral cavity, T2N0M0	Postoperative RT, 2 Gy × 33, 5 F/W	0	0	59	98
OW5	63	M	Living alone, unemployed	Pharynx, T4aN2cM0	RT, 2 Gy × 34, 6 F/W	0	0	10	17
OW1	60	M	Living alone, retired	Oral cavity, T4aN0M0	Postoperative CRT, 2 Gy × 33, 6 F/W	0	0	9	15

Wear time > 12 h/d: number of days wherein the smartwatch was worn >12 h/d; wear time %: percentage of time the smartwatch was worn >12 h/d during a study period of 60 days; data acquisition days: number of days with any data registration; data acquisition %: percentage of data acquisition during a study period of 60 days. CRT: chemoradiotherapy; RT: radiotherapy; F/W: fractions per week.

## Data Availability

The data presented in this study can be obtained upon reasonable request and if in agreement with institutional restrictions.

## Supplementary Materials

The following supporting information can be downloaded at https://www.mdpi.com/article/10.3390/cancers15020422/s1. Figure S1—examples of heart rate and step count variations during the study period: (a) patient ID OW2; (b) patient ID OW9.
