# Supplementary material for: Feasibility of Using Wearables for Home Monitoring during Radiotherapy for Head and Neck Cancer—Results from the OncoWatch 1.0 Study
Source: Cancers (Basel). 2023 Jan 9;15(2):422. doi: 10.3390/cancers15020422 (PMC9857313; doi:10.3390/cancers15020422)
Supplement: Supplementary file 1 [file cancers-15-00422-s001.zip › cancers-2096928-supplementary.pdf]

Supplementary Figure S1

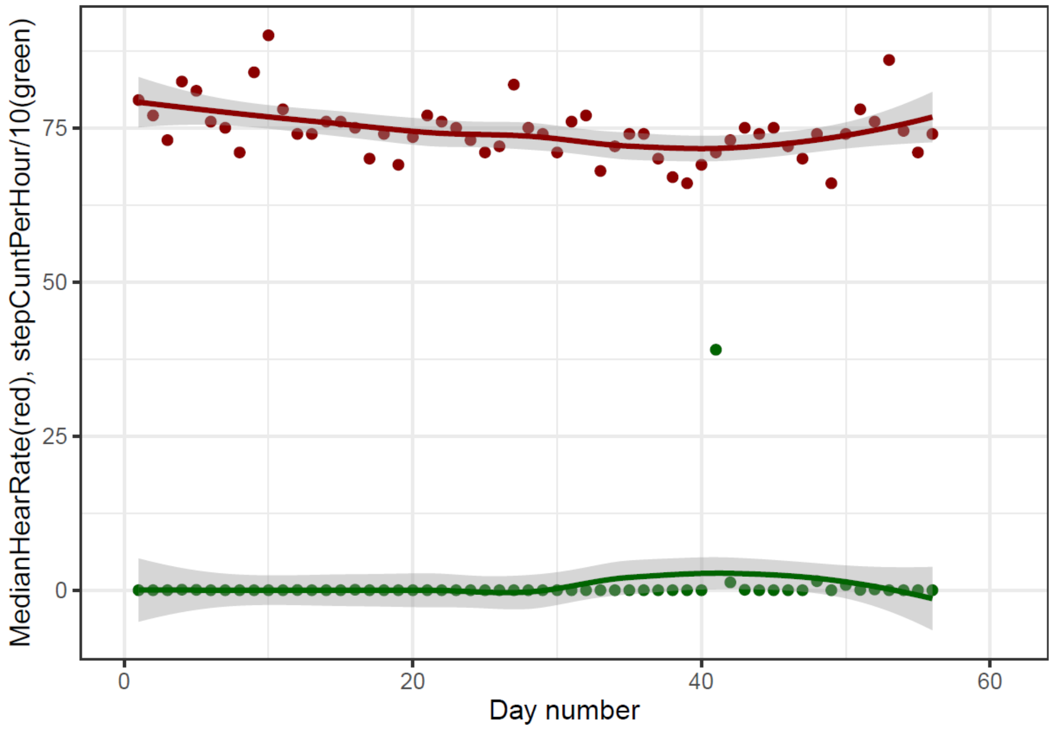

(a)

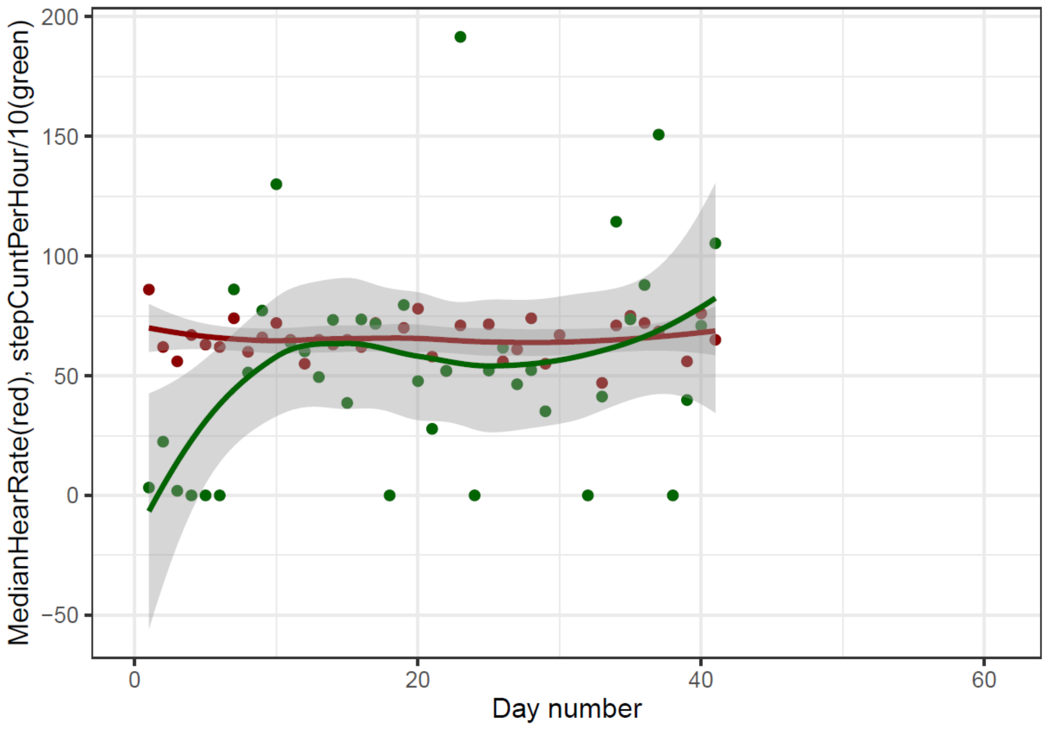

(b)

Examples of heart rate and step count variations during the study period: (**a**) Patient ID OW2 ; (**b**) Patient ID OW9.
